# Association Between Thyroid-Stimulating Hormone and Estimated Glomerular Filtration Rate in the Third Trimester of Pregnancy: A Retrospective Cross-Sectional Study in Euthyroid Women

**DOI:** 10.3390/medicina61112046

**Published:** 2025-11-16

**Authors:** Canan Satır Özel, Yaşar Sertbaş, Şeyma Taştekin, Asya Tancer Özçelik, Meltem Sertbaş, Özge Kınlı Yıldız, Abdulkadir Turgut

**Affiliations:** 1 Department of Obstetrics and Gynecology, Faculty of Medicine, Istanbul Medeniyet University, Istanbul 34722, Turkey; seytastekin@gmail.com (Ş.T.); abdulkadirturgut@gmail.com (A.T.); 2 Department of Internal Medicine, Fatih Sultan Mehmet Education and Research Hospital, Istanbul 34752, Turkey; yserzincan24@gmail.com (Y.S.); msertbas68@gmail.com (M.S.); 3 Department of Obstetrics and Gynecology, Faculty of Medicine, Pamukkale University, Denizli 20070, Turkey; drtancer@hotmail.com; 4 Department of Obstetrics and Gynecology, Faculty of Medicine, Bahçeşehir University, İstanbul 34732, Turkey; drozgekinliyildiz@gmail.com

**Keywords:** pregnancy–third trimester, euthyroid state, thyrotropin, triiodothyronine, glomerular filtration rate, kidney function test

## Abstract

*Background and Objectives*: This study investigated the relationship between thyroid function and renal parameters during the third trimester of pregnancy in euthyroid women, a physiological interaction that remains poorly characterized. *Materials and Methods*: In this retrospective, single-center cross-sectional study, 820 euthyroid pregnant women (≥28 weeks of gestation) were evaluated. Thyroid-stimulating hormone (TSH), free triiodothyronine (fT3), free thyroxine (fT4), serum creatinine, and estimated glomerular filtration rate (eGFR) were analyzed using tertile-based comparisons, correlation tests, and linear regression analysis. *Results*: Higher TSH levels were associated with slightly higher serum creatinine (*p* = 0.011) and a weak negative correlation with eGFR (r = −0.079, *p* = 0.023). Conversely, fT3 levels were positively correlated with eGFR (r = 0.106, *p* = 0.002) and inversely correlated with creatinine (r = −0.074, *p* = 0.035), while fT4 showed weaker associations. Regression analysis confirmed that fT3 (β = 0.099, *p* = 0.005) and fT4 (β = 0.083, *p* = 0.019) were independent positive predictors of eGFR. *Conclusions*: The correlations observed were statistically significant but clinically modest. Regression analysis confirmed that FT3 and FT4 were independent positive predictors of GFR, suggesting that subtle variations in thyroid activity may reflect physiological rather than pathological renal adaptations in late pregnancy. Monitoring TSH and fT3 may enhance understanding of maternal endocrine and renal interplay, though the clinical utility of such associations remains limited and warrants confirmation in prospective studies.

## 1. Introduction

Pregnancy induces profound biological, hormonal, and metabolic adaptations, with the maternal endocrine and renal systems playing pivotal roles in meeting maternal and fetal demands. The dynamic changes in thyroid hormone levels and renal function are integral components of these physiological adaptations [1].

Elevated maternal estradiol increases hepatic synthesis and decreases degradation of thyroid hormone-binding globulin (TBG), resulting in a 1.5–2-fold rise in TBG concentrations and consequently higher total T4 and T3 levels, which elevate thyroid hormone requirements [2,3,4]. Placental type 3 deiodinase further enhances thyroid hormone demand by converting T4 and T3 into their inactive metabolites, whereas high human chorionic gonadotropin (hCG) concentrations stimulate thyroid hormone production via TSH receptor activation, leading to physiological TSH suppression during early gestation [1,3]. As hCG levels decline after the first trimester, TSH levels rise, underscoring the importance of trimester-specific reference ranges for thyroid assessment [4,5].

In parallel, systemic vasodilation, increased cardiac output, and plasma volume expansion markedly augment renal plasma flow and glomerular filtration rate (GFR), which increase by approximately 40–60% by mid-pregnancy [6,7]. These endocrine and hemodynamic adjustments, supported by the renin–angiotensin–aldosterone system and placental deiodinase activity, are essential for maintaining maternal–fetal homeostasis [8,9].

Thyroid hormones exert broad effects on renal physiology by modulating cardiac output, vascular resistance, and glomerular hemodynamics. In hyperthyroidism, renal plasma flow and GFR rise by 20–30%, whereas hypothyroidism is associated with reduced GFR and elevated creatinine levels [10,11]. Even within the euthyroid range, subtle variations in TSH have been shown to influence renal blood flow and filtration dynamics [12].

Despite well-documented thyroid–renal interactions in general populations, data on these relationships during pregnancy remain scarce. This study aimed to investigate how maternal thyroid activity influences renal parameters in euthyroid women. We hypothesized that even subtle variations in TSH, fT3, and fT4 levels within the reference range are associated with measurable changes in renal function markers, particularly eGFR and serum creatinine, during the third trimester. Understanding these associations may provide insight into maternal endocrine–renal adaptation and support improved perinatal monitoring strategies.

## 2. Materials and Methods

### 2.1. Study Design and Setting

This retrospective, single-center cross-sectional study was conducted at a tertiary care hospital (the Department of Obstetrics and Gynecology, Goztepe Prof. Dr. Suleyman Yalcın City Hospital) between 1 January and 31 December 2021. The study was approved by the institutional review board (2022/0138), and all procedures adhered to the Declaration of Helsinki.

### 2.2. Participants and Data Collection

Medical records of 1374 pregnant women admitted to the delivery unit during the study period were reviewed. All laboratory assessments, including thyroid and renal function parameters, were performed during the third trimester, specifically between the 28th and 40th weeks of gestation, as part of routine antenatal evaluation. Among them, 985 had complete laboratory data including thyroid-stimulating hormone (TSH), free triiodothyronine (fT3), free thyroxine (fT4), serum creatinine, and estimated glomerular filtration rate (eGFR), as well as demographic and clinical characteristics.

Inclusion Criteria:

- Maternal age between 18 and 45 years;
- Gestational age ≥ 28 weeks (third trimester);
- Euthyroid status, defined as TSH, fT3, and fT4 levels within the reference ranges.

Exclusion Criteria:

- Maternal age < 18 or >45 years;
- Gestational age < 28 weeks;
- Any form of thyroid dysfunction (subclinical or overt hypothyroidism or hyperthyroidism);
- Hypertensive disorders;
- Gestational or pregestational diabetes mellitus;
- Chronic kidney disease;
- Muscular or urinary tract disorders.

**Figure 1:**
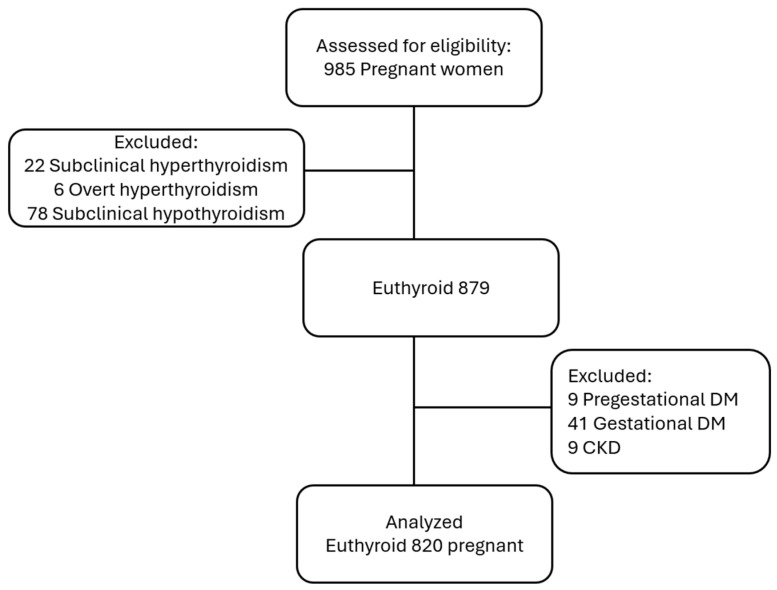
Flow diagram illustrating the selection process and exclusion criteria applied to identify the final study cohort of euthyroid pregnant women.

Participants were screened according to these predefined criteria, and those not meeting the inclusion requirements or meeting any exclusion criteria were systematically removed from the analysis. A total of 106 women were excluded due to thyroid dysfunction: 22 with subclinical hyperthyroidism, 6 with overt hyperthyroidism, and 78 with subclinical hypothyroidism. Of the remaining 879 euthyroid women, 59 were excluded due to comorbid conditions, including pregestational diabetes mellitus (n = 9), gestational diabetes mellitus (n = 41), and chronic kidney disease (n = 9). Ultimately, 820 euthyroid pregnant women were included in the final analysis. Flow diagram of participant selection is shown in Figure 1.

### 2.3. Sample Size

Although this was a retrospective study including all eligible euthyroid pregnant women (n = 820), a post hoc power analysis confirmed that the sample size was adequate to detect small but meaningful correlations (expected r = 0.10) with >80% power at a two-sided α = 0.05. The minimum required sample size for this effect was approximately 782, confirming that our final cohort (n = 820) provided sufficient statistical power to detect weak correlations between thyroid and renal parameters.

### 2.4. Laboratory Measurements

Serum TSH, fT4, and fT3 levels were measured using the Roche Cobas e801 Immunoassay Analyzer (Roche Diagnostics, Mannheim, Germany) employing the Electrochemiluminescence Immunoassay (ECLIA) method. The reference ranges were 0.54–4.31 mIU/L for TSH, 0.93–1.71 ng/dL for fT4, and 2.04–4.4 pg/mL for fT3.

Serum creatinine was analyzed with the Roche Cobas c702 Clinical Chemistry Analyzer (Roche Diagnostics, Mannheim, Germany) using the Kinetic Colorimetric Jaffe method, with a reference range of 0.5–0.9 mg/dL.

Estimated glomerular filtration rate (eGFR) was automatically calculated through the ALIS Laboratory Information Management System using the CKD-EPI (Chronic Kidney Disease Epidemiology Collaboration) equation [13]. Although the CKD-EPI formula may slightly overestimate eGFR during pregnancy due to physiological hyperfiltration, it remains the most validated and widely used method for clinical estimation of renal function [14].

Women were classified as euthyroid if all three thyroid parameters were within the respective reference ranges. In third-trimester euthyroid women, TSH, fT3, and fT4 levels were stratified into tertiles, and their associations with eGFR and serum creatinine were evaluated, consistent with previous studies [14,15].

### 2.5. Statistical Analysis

All statistical analyses were conducted using IBM SPSS Statistics, version 22.0 (IBM Corp., Armonk, NY, USA). The normality of continuous variables was assessed using the Kolmogorov–Smirnov test and by visual inspection of histograms. Variables with a normal distribution were reported as mean ± standard deviation (SD), whereas those with a non-normal distribution were presented as median and interquartile range (IQR).

To compare renal function parameters across tertiles of thyroid hormone levels (TSH, fT3, fT4), one-way analysis of variance (ANOVA) was used for normally distributed variables, and the Kruskal–Wallis test was applied for non-normally distributed variables. When the Kruskal–Wallis test indicated significant differences among tertiles, pairwise comparisons were subsequently performed using the Mann–Whitney U test with Bonferroni-adjusted *p*-values.

Associations between thyroid hormones and renal function parameters were evaluated using Pearson correlation coefficients for normally distributed variables and Spearman’s rank correlation coefficients for non-parametric variables.

In addition, a linear regression analysis was performed to determine the independent effects of thyroid function parameters (TSH, fT3, and fT4) on estimated glomerular filtration rate (eGFR). The regression model included eGFR as the dependent variable and thyroid parameters as independent predictors. Model fit was evaluated using the coefficient of determination (R^2^) and the F-test.

A two-tailed *p*-value < 0.05 was considered statistically significant for all analyses.

## 3. Results

A total of 985 pregnant women were initially assessed for eligibility. Among them, 106 were excluded due to abnormal thyroid function, including 22 with subclinical hyperthyroidism, 6 with overt hyperthyroidism, and 78 with subclinical hypothyroidism, leaving 879 euthyroid pregnant women eligible for further evaluation.

Of these, an additional 59 were excluded due to comorbidities: 9 had pregestational diabetes mellitus, 41 had gestational diabetes mellitus, and 9 had chronic kidney disease. Consequently, 820 euthyroid pregnant women were included in the final analysis (Figure 1).

A total of 820 third-trimester euthyroid pregnant women met the study criteria and were included in the analysis. Baseline demographic and clinical characteristics of the participants are summarized in Table 1.

Associations between thyroid function tertiles and renal function parameters are presented in Table 2. Overall analysis showed a significant difference in serum creatinine levels across TSH tertiles (*p* = 0.011), and in eGFR values across both FT3 (*p* = 0.034) and FT4 (*p* = 0.038) tertiles. Post hoc Mann–Whitney U tests demonstrated that creatinine levels were significantly higher in the second and third TSH tertiles compared with the first (*p* = 0.008 and *p* < 0.001, respectively), while no difference was found between the second and third tertiles (*p* = 0.445). For fT3, eGFR was significantly higher in the third tertile compared with the first (*p* = 0.001), whereas other comparisons were not significant (*p* > 0.05). For fT4, eGFR was higher in the third tertile compared with both the first (*p* = 0.030) and the second tertiles (*p* = 0.045), with no difference between the first and second tertiles (*p* = 0.631).

Collectively, these findings indicate that higher fT3 and fT4 levels are associated with slightly increased eGFR, whereas higher TSH levels correspond to modest elevations in serum creatinine, suggesting subtle physiological variations in renal function within the euthyroid range.

**Figure 2:**
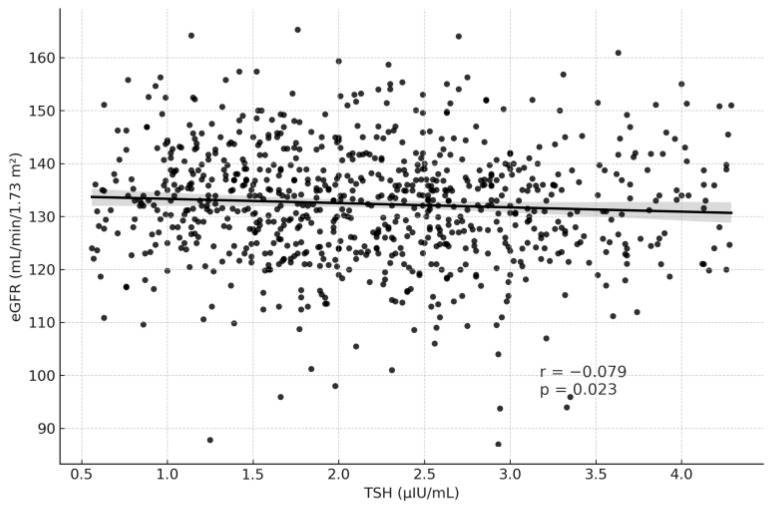
Correlation Graph of TSH and eGFR.

**Figure 3:**
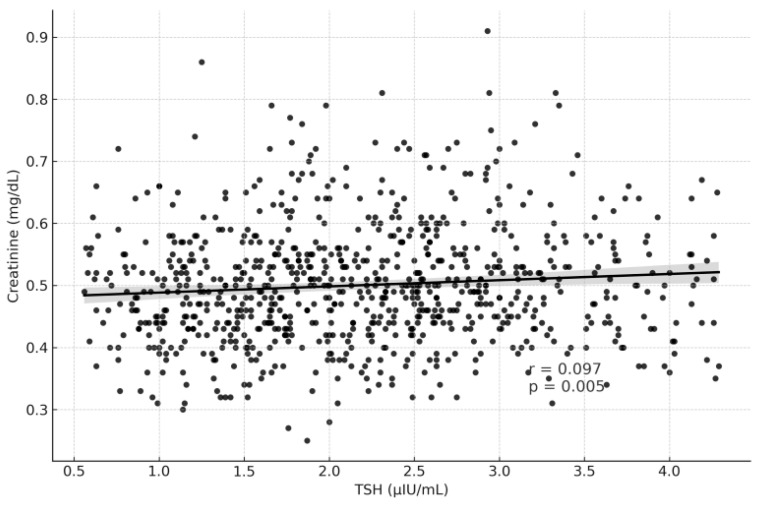
Correlation Graph of TSH and Creatinine.

**Figure 4:**
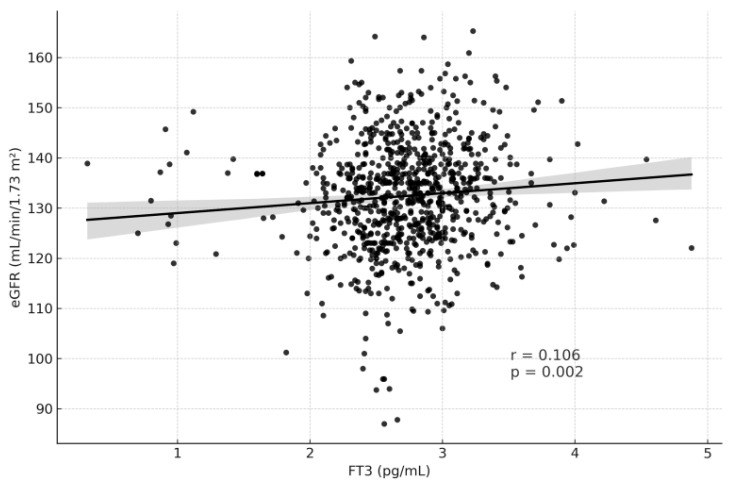
Correlation Graph of fT3 and eGFR.

**Figure 5:**
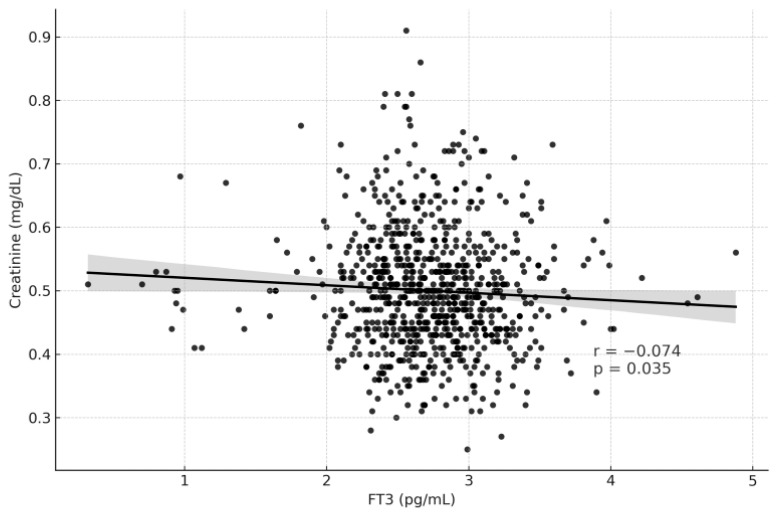
Correlation Graph of fT3 and Creatinine.

Table 3 summarizes the correlation analysis between thyroid function tests and renal function parameters. TSH levels showed weak but statistically significant correlations, being positively associated with serum creatinine (r = 0.097, *p* = 0.005) and negatively associated with eGFR (r = −0.079, *p* = 0.023) (Figure 2 and Figure 3). In contrast, fT3 demonstrated weak but significant positive correlations with eGFR (r = 0.106, *p* = 0.002) and negative correlations with creatinine (r = −0.074, *p* = 0.035) (Figure 4 and Figure 5). fT4, however, did not show statistically significant correlations with either renal function marker.

In a linear regression model with eGFR as the dependent variable and TSH, fT3, and fT4 as predictors, the overall model was statistically significant (F = 4.945, *p* = 0.002, R^2^ = 0.018). Among thyroid parameters, fT3 (β = 0.099, *p* = 0.005) and fT4 (β = 0.083, *p* = 0.019) were significant positive predictors of eGFR, whereas TSH showed a negative but non-significant association (β = −0.064, *p* = 0.067). These findings suggest that higher fT3 and fT4 levels are independently associated with higher glomerular filtration rates, even within the euthyroid range, whereas elevated TSH tends to be linked with slightly reduced renal function (Table 4).

## 4. Discussion

In this cross-sectional study of 820 euthyroid pregnant women, we identified subtle but statistically significant associations between thyroid function parameters and renal function indices. Although all thyroid hormone levels remained within the physiological reference range, higher TSH and lower fT3 concentrations were linked to mild alterations in serum creatinine and estimated glomerular filtration rate (eGFR). These findings provide new evidence that thyroid–renal crosstalk operates even in the absence of overt thyroid dysfunction, reflecting the sensitivity of renal hemodynamics to minor endocrine fluctuations during pregnancy—a period of profound metabolic and circulatory adaptation [5,6].

Consistent with previous population-based studies, we observed a weak inverse correlation between TSH and eGFR (r = −0.079, *p* = 0.023), and a positive correlation between fT3 and eGFR (r = 0.106, *p* = 0.002). Although the effect sizes were small, these results align with the findings of Sun et al. and Asvold et al., who demonstrated that TSH levels within the reference range are inversely related to renal filtration capacity [15,16]. Physiologically, TSH may influence renal perfusion through modulation of systemic vascular resistance and intraglomerular pressure. Tsuda et al. [12] reported that elevated TSH increases afferent arteriolar resistance and reduces renal plasma flow, offering a plausible mechanism that could be amplified by the hemodynamic demands of pregnancy.

Pregnancy itself is characterized by marked renal vasodilation, plasma volume expansion, and glomerular hyperfiltration beginning in early gestation [5,6]. These hemodynamic shifts are hormonally driven and supported by activation of the renin–angiotensin–aldosterone system, endothelial nitric oxide pathways, and placental deiodinase activity, which modulates maternal thyroid hormone bioavailability [1,2,5,8,9]. Recent obstetric literature further emphasizes that placental type 3 deiodinase expression increases local T3 degradation, thereby influencing maternal–fetal thyroid balance and potentially affecting renal hemodynamic responses [5,8,9]. Within this context, even minor variations in thyroid activity may subtly modify glomerular filtration or renal plasma flow. Our results suggest that low–normal fT3 levels and higher TSH concentrations could indicate suboptimal maternal adaptation to these physiological changes, potentially reflecting altered endothelial responsiveness or systemic vascular tone.

The linear regression analysis further supported these associations. The model was statistically significant (F = 4.945, *p* = 0.002; R^2^ = 0.018), showing that fT3 (β = 0.099, *p* = 0.005) and fT4 (β = 0.083, *p* = 0.019) were independent positive predictors of eGFR, while TSH displayed a negative but non-significant trend (β = −0.064, *p* = 0.067). Although the explained variance was modest, these findings reinforce that thyroid hormones exert a measurable, independent influence on renal function even within reference limits.

The associations we observed are consistent with prior research in non-pregnant populations. Keskin et al. identified TSH as a negative predictor of eGFR in euthyroid individuals with metabolic syndrome, and Wang et al. reported an 18% higher risk of diabetic nephropathy for each one–standard deviation increase in TSH among euthyroid patients with type 2 diabetes [17,18]. Similarly, the ELSA-Brasill study by Peixoto de Miranda et al. demonstrated an inverse association between TSH and eGFR in healthy adults [19]. Conversely, Patil et al. found no significant associations, emphasizing that these relationships may vary depending on population context and underlying metabolic status [20]. Overall, the preponderance of evidence including our data suggests that even small variations in thyroid hormones may modestly influence renal function in biochemically euthyroid individuals.

Regarding thyroid hormones, our findings revealed that higher fT3 levels were associated with higher eGFR (r = 0.106, *p* = 0.002) and lower serum creatinine (r = −0.074, *p* = 0.035), whereas fT4 exhibited a weaker but significant relationship with eGFR (*p* = 0.038). These results are consistent with previous studies by Liu et al. and Anderson et al., both of which demonstrated that fT3 but not TSH or fT4 is independently associated with renal filtration indices [11,21]. Additionally, Zhang et al. showed that fT3 levels below 3 pg/mL increased the risk of chronic kidney disease in euthyroid adults, reinforcing the hypothesis that even subtle reductions in fT3 may reflect early renal dysfunction or impaired systemic metabolism [22].

From a clinical standpoint, the observed correlations were statistically significant yet clinically small. Although the observed correlations were weak, they may represent subtle physiological interactions rather than strong predictive markers. These findings should therefore be interpreted as physiological rather than pathological, suggesting early indicators of endocrine–renal interaction rather than markers of overt disease. The absence of maternal or neonatal outcomes limits direct clinical interpretation, and future prospective studies are warranted. Nonetheless, our findings underscore a potentially overlooked mechanism linking thyroid status and renal hemodynamics during pregnancy, warranting further longitudinal exploration.

Several limitations should be noted. First, the retrospective, single-center design may introduce selection bias and limit control for confounders such as BMI, parity, anemia, nutritional, and iodine status. Second, trimester specific reference ranges for thyroid hormones were not available, and fixed laboratory cut-offs were used. Third, the CKD-EPI equation, although widely validated, may overestimate eGFR in pregnancy due to physiological hyperfiltration [9]. Finally, the cross-sectional design precludes causal inference, and future prospective studies incorporating maternal and neonatal outcomes are necessary to clarify the long-term implications of these findings.

### Clinical Implications

Clinically, these findings suggest that subtle variations in thyroid function, even within normal limits, may contribute to renal adaptation during late pregnancy. Routine thyroid function assessment particularly of TSH and fT3 levels may provide additional insight into maternal renal hemodynamics. Integrating thyroid and renal parameters into antenatal follow-up could enhance early recognition of subclinical renal stress and improve understanding of maternal–fetal physiological adaptation.

## 5. Conclusions

Taken together, our findings demonstrate that higher TSH and lower fT3 levels, even within reference limits, are associated with mild alterations in renal function during pregnancy. Linear regression analysis further indicated that fT3 and fT4 were independent positive predictors of eGFR, suggesting a modest but measurable endocrine influence on renal filtration. These results highlight an underrecognized endocrine–renal interaction with physiological significance in maternal adaptation to pregnancy. Future longitudinal studies are needed to determine whether these subtle hormonal variations influence pregnancy outcomes or long-term renal health.

## Figures and Tables

**Table 1:** Baseline Characteristics of the Study Population.

Variable	Mean ± SD
Age (years)	28.66 ± 6.02
TSH (μIU/mL)	2.18 ± 0.86
FT3 (ng/L)	2.72 ± 0.47
FT4 (ng/dL)	0.98 ± 0.23
eGFR (mL/min/1.73 m^2^)	132.36 ± 10.96
Creatinine (mg/dL)	0.50 ± 0.09
Gravida (n)	2.53 ± 1.57
Gestational age (weeks)	38.69 ± 2.21
Birth Weight (g)	3199.67 ± 588.75
Birth Height (cm)	49.76 ± 22.85
Neonatal Head Circumference (cm)	34.86 ± 2.5
Cesarean Section Rate	487 (49.4%)
Apgar 1	7.33 ± 1.76
Apgar 5	9.04 ± 1.70

**Table 2:** Relationship Between Thyroid Function Tests and Renal Function Parameters Across Tertiles.

		eGFR (mL/min/1.73 m^2^)	*p*	Creatinine (mg/dL)	*p*
TSH	1. Tertile median (IQR)	133 (13)	0.187	0.47(0.13)	0.011
	2. Tertile median (IQR)	132 (14)		0.50 (0.06)	
	3. Tertile median (IQR)	131 (14)		0.50 (0.13)	
fT3	1. Tertile median (IQR)	131 (14)	0.034	0.51 (0.12)	0.066
	2. Tertile median (IQR)	132 (14)		0.49 (0.12)	
	3. Tertile median (IQR)	133 (14)		0.48 (0.08)	
fT4	1. Tertile median (IQR)	131 (14)	0.038	0.49 (0.12)	0.633
	2. Tertile median (IQR)	132 (14)		0.49 (0.11)	
	3. Tertile median (IQR)	134 (12)		0.50 (0.11)	

TSH: thyroid-stimulating hormone; fT3: free triiodothyronine; fT4: free thyroxine, all comparisons between tertiles were performed using the Kruskal–Wallis test.

**Table 3:** Correlations Between Thyroid Function Tests and Renal Function Parameters.

Variable	eGFR (mL/min/1.73 m^2^)		Creatinine (mg/dL)	
	r	*p*-Value	r	*p*-Value
TSH (μIU/mL)	−0.079	0.023	0.097	0.005
fT3 (ng/L)	0.106	0.002	−0.074	0.035
fT4 (ng/dL)	0.067	0.058	0.045	0.196

TSH: thyroid-stimulating hormone; fT3: free triiodothyronine; fT4: free thyroxine. All correlations were analyzed using Spearman’s rank correlation test.

**Table 4:** Linear regression analysis evaluating the association between thyroid function parameters and estimated glomerular filtration rate (eGFR).

Predictor	B	SE	β	t	*p*-Value
Constant	124.044	3.180	—	39.009	<0.001
TSH (0.5–5.1 mIU/L)	−0.811	0.441	−0.064	−1.837	0.067
fT3 (2.04–4.4 ng/L)	2.316	0.830	0.099	2.790	0.005
fT4 (0.93–1.71 ng/dL)	3.901	1.659	0.083	2.351	0.019

## Data Availability

The original contributions presented in this study are included in the article. Further inquiries can be directed to the corresponding author.
